# Finding digital health governance mechanism to support country’s health systems: Thailand case study

**DOI:** 10.1093/oodh/oqae019

**Published:** 2024-06-12

**Authors:** Boonchai Kijsanayotin, Anawat Ratchatorn, Kamonporn Suwanthaweemeesuk

**Affiliations:** Department of Clinical Epidemiology and Biostatistics, Faculty of Medicine Ramathibodi Hospital, Mahidol University, Sukhothai Road, Dusit District, Bangkok 10300, Thailand; Faculty of Tropical Medicine, Mahidol University, Ratchawithi Road, Ratchathewi District, Bangkok 10400, Thailand; Chakri Naruebodindra Medical Institute, Faculty of Medicine Ramathibodi Hospital, Mahidol University, Suwannabhumi Canal Road, Bang Phli, Samut Prakan 10540, Thailand

**Keywords:** digital health governance, health systems, Thailand, policy recommendation, eHealth, health information standards

## Abstract

In 2010, a collaboration between the Ministry of Public Health and the World Health Organization Thailand highlighted the urgent need for an effective eHealth governance mechanism in the country. Despite efforts, a consensus-driven governance mechanism remains elusive. This research aimed to investigate suitable digital health governance models for Thailand by examining models from six countries (Malaysia, the Philippines, Australia, England, the USA and Canada) and gathering insights from stakeholders. In stage 1, research gathered data via literature reviews and interviews with 11 executives in Thailand’s digital health sectors. The study of six countries showed diverse digital health governance influenced by political, cultural and health factors. Using the Broadband Commission’s governance models, most participants preferred a dedicated digital health agency. They emphasized decisive leadership, collaboration to prevent silos and uniform health information standards. In Thailand, the Ministry of Public Health cannot oversee digital health solely but can lead in tandem with other bodies. Effective governance requires collaboration, leadership and the dedicated agency model, underscoring health information standards’ significance. Stage 2 published the ‘Digital Health Governance Model: Recommendation for Thailand Health Systems’, presented to 101 high-level representatives. A survey indicated that over 90% of these stakeholders concurred with the study’s findings and recommendations. The research suggests that while the Ministry of Public Health is central, it should not manage alone. Collaborative governance with consistent leadership is crucial for Thailand’s digital health progression. Although the study lacked civil society input, its insights are pivotal for Thailand’s digital health policy future.

## Lay Summary

Thailand has recognized the importance of improving its digital health systems. To achieve this, a study was conducted to find the best way to manage these systems effectively. The research involved examining digital health management in six countries—Malaysia, the Philippines, Australia, England, the USA and Canada—to understand different approaches to overseeing such systems.

The first phase of the study involved interviews with key people from both the government and private sectors in Thailand to determine which management model would be most suitable for the country. The findings highlighted the need for a separate agency dedicated to digital health, strong leadership, effective teamwork and consistent standards for health information across Thailand. The study showed that while the Ministry of Public Health (MOPH) in Thailand is crucial, it should work together with other organizations for better management.

In the second phase, a proposed model for managing Thailand’s digital health was presented to Thai health officials and experts. A survey conducted among these professionals showed that most agreed with the proposed model. This study underscores the need for collaborative efforts and strong, consistent leadership to advance Thailand’s digital health systems, ensuring better health care for everyone.

## INTRODUCTION

In 2010, the Ministry of Public Health (MOPH), in partnership with the World Health Organization (WHO) Thailand, researched the state of health information systems, health information and communication technology, or e-Health in Thailand (in this study we use digital health and eHealth interchangeably). The study identified a significant challenge which is the underdeveloped foundational elements of the national health information systems [1]. The report stressed the need for Thailand to prioritize these components, particularly emphasizing the establishment of an eHealth governance mechanism. Such a mechanism should engage relevant stakeholders and bolster the country’s eHealth and broader health systems’ efficacy.

Since then, many organizations have attempted to establish a mechanism to oversee the digital health. However, this has not yet been realized due to the inability to reach a consensus among the involved parties. Hence, this study aims to explore digital health governance models from different countries, assess Thailand’s current situation and suggest policy options fitting Thailand’s health system context [2].

## MATERIALS AND METHODS

**Figure 1:**
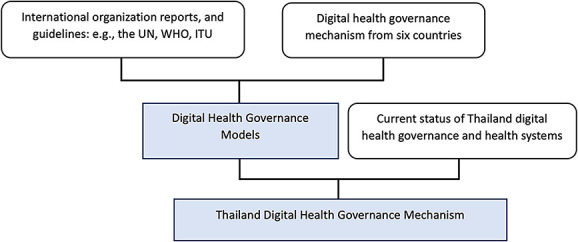
The study conceptual model **(**UN = United Nations, WHO = World Health Organization, ITU = International Telecommunication Union)

**Stage 1:** We collated information on health systems and digital health governance from various documents and reports, focusing on Thailand and six other countries: Malaysia, the Philippines, Australia, England, the USA and Canada. Additionally, guidelines and studies from international organizations, notably the United Nations (UN), WHO and the International Telecommunication (ITU), were reviewed. Data were also sourced from in-depth interviews with executives from the MOPH and outside the ministry, academics and private sector entrepreneurs, a total of 11 people. For the sample selection method, we started by identifying three policy-making level informants one from the MOPH, one from academia and one from private sector, and then employed the snowball sampling technique, asking the informants to help identify the next informants. The interviews, both face-to-face and online, took a total of 615 min, averaging 56 min per person [2] (Fig. 1, Textbox 1).

**Textbox 1:** In-depth interview process and semi-structured questions for the digital health stakeholder informants

The researcher introduces themselves and the research project. The researcher interviews the informant using semi-structured questions, which include: Work experience in digital health. Understanding of the term ‘governance’. Opinion on the current situation of digital health in Thailand. Aspects of digital health in Thailand that are positive and those that need improvement. Types of digital health governance system most suitable for Thailand. Opinion on how to implement the model and the challenges involved. Who or which agency should lead the implementation of this model.

**Stage 2:** From the literature review and in-depth interviews, we derived insights and published t*he ‘Digital Health Governance Model: Recommendation for Thailand Health Systems’ report* [2]. We distributed the report and presented the study findings and policy recommendations to 101 high level administrators and representatives of Thailand digital health stakeholders at the ‘Solutions to the challenges and obstacles in the development of digital health in Thailand’ seminar organized by the Digital Health Subcommittee of the Public Health Committee of the Senate. The participants comprised administrators from various departments and semi-governmental agencies from MOPH, Ministry of Digital Economy and Society, Ministry of Higher Education, Science, Research and Innovation, Ministry of Education, national health insurance agencies, health professional councils, public and private hospitals and the senators. We conducted the survey asking the degree of agreement and opinions regarding the study finding and recommendations. The questionnaire employed Likert scale measure from 1 to 5 where 5 = strongly agreed, 4 = agreed, 3 = neutral, 2 = disagreed and 1 = strongly disagreed.

## RESULTS

### Stage 1

#### Definition of governance and digital health governance

In this study, ‘digital health governance’ refers to the mechanisms and processes of decision-making for the implementation (or non-implementation) of various activities of the digital health. The process involves exercising ‘power’ to set digital health directions, allocating limited resources and assessing risks arising from the implementation (or non-implementation). Moreover, it involves processes that are related and have impacts on stakeholders involved in the decision-making process, both formal and informal, including government agencies, the private sector and communities. Its implications extend beyond state authority. In other words ‘governance’ is not ‘government’ [2].

Digital health governance requires collaboration among various state agencies, the private sector, educational institutions, professional associations and communities. It is a process that manages, controls, supports, coordinates, establishes ownership and accountability of digital health systems together [2].

Furthermore, ‘Digital health governance’ is different from ‘digital health data governance’, the former encompasses broader aspects of health systems while the latter specifically addresses decision-making related to health data.

The UN has proposed the eight elements of ‘good governance’ [3]: 1. Participation, 2. Rule of law, 3. Consensus orientation, 4. Equity and inclusiveness, 5. Effectiveness and efficiency, 6. Accountability, 7. Transparency, and 8. Responsiveness.

#### Digital health governance mechanism

Upon examination of the governance mechanisms for digital health in various countries, we can identify and differentiate models of digital health governance by considering the agencies that play a significant role in the development and driving of the digital health system. We found three models [4] (Table 1).

**Table 1:** Three types of digital health governance mechanism (adapted from ITU-Broadband Commission 2017 report) [4]

	Ministry of Health(MOH) mechanism	Government-wide digital agency mechanism	Dedicated digital health agency mechanism
Operational model	MOH is responsible for driving the digital health project by procuring resources, capabilities and technical skills from the ministry, private agencies and other organizations.	The government promotes digital development nationwide, establishing a national digital architecture. The MOH advances digital health based on central government guidelines and IT infrastructure.	MOH leads the development of digital health strategies and guides digital health strategies, supports external digital health units under its purview and coordinates with other Ministries and agencies.
Strengths	MOH understands key health issues, ensuring confident planning, clear stakeholder roles and minimized sectoral conflicts.	Governmental organizations follow uniform national enterprise architecture, data standards and guidelines, promoting collaboration and joint investments between agencies, thereby reducing costs.	Jointly designed health data architectures ensure consistent information exchange across units, maintaining objectives even with leadership changes, and fostering technical knowledge advancement.
Challenges	The sustainability and evolution of digital health systems when there is a leadership change, and unable to change as rapid as the change of technology.	Consolidating and aligning responsibilities at the central level and coordinating efforts between the MOH, the Ministry of ICT and other Ministries.	Building the credibility of specialized agencies depends on transparency, and there might be misaligned needs between specific project focuses and overall necessities.
Country examples	Brazil, Chile, Ghana, Kenya, Malawi, Philippines, Rwanda, South Africa.	Malaysia, Estonia, Singapore, Bangladesh, Uruguay	Canada, Mali, Norway, Australia and England are also referenced.

Digital health governance varies by country, influenced by political systems, health systems, culture and national contexts. Digital health governance in each country spans a spectrum from a centralized approach to a more decentralized, local governance model. Consequently, some countries exhibit a combination of the three identified models (Table 2).

**Table 2:** Health systems and digital health governance mechanism of the six studied countries

Country and its digital health governance mechanism	Health system	Digital health governance
**Australia** Dedicated digital health agency mechanism.	Australia’s health system consists of three levels [5]: 1. The national government funds states and oversees health insurance. 2. State governments manage hospitals, public health and emergencies. 3. Local governments handle community health and preventive care.	The Australian Digital Health Agency is a corporate entity aiming to enhance public health through technology and innovation, funded mainly by the national government and partially by states. The 2016 National Digital Health Strategy involved collaboration with the public sector, service providers, researchers and the industry [6–8].
**Philippines** Health ministry mechanism.	The Philippines’ health system combines public and private sectors. Public health services, funded by taxes, include [9]: 1. The national government overseeing administration, policies, standards and national hospitals. 2. Local governments managing local health services, hospitals and health promotion.	The Ministries of Health, Science and Technology, and the National Health Insurance Company collaborate in their respective areas. The digital health system is directed by the National eHealth Steering Committee and the Technical Working Group. The MOH leads the Steering Committee, guiding, assessing, and supervising the Technical Working Group, which works based on specific frameworks and plans. The country’s digital health governance adheres to the WHO-ITU National eHealth Strategy Toolkit [4, 10].
**USA** Dedicated digital health agency mechanism + Health ministry mechanism	The US health system is very complex due to diverse health insurance systems and medical service systems that often do not coordinate with each other. Health administration is under the responsibility of the US Department of Health and Human Services. The main components of the US public health system are [11]: 1. Government, 2. Private insurance, 3. Health service providers and 4. Regulators	The Office of the National Coordinator for Health Information Technology (ONC) promote the standardized use and exchange of secure health information. It has received short-term funding for initial projects, such as the Medicare and Medicaid reimbursement programs through electronic health records. ONC coordinates high-level government and private sector initiatives, supports quality assurance projects and develops related policies. [12, 13]
**Canada** Dedicated digital health agency mechanism	Canada’s health system provides free medically necessary services to all citizens through a ‘state-paid, private-service’ model. Each region has its insurance plan under ‘Medicare’. The core principles of Canada’s Medicare are [14]: 1. Public administration, 2. Comprehensiveness, 3. Universality, 4. Accessibility and 5.Portability.	Canada Health Infoway is a non-profit that promotes digital health connectivity. Funded by the federal government and partnered with local governments, each provincial/territorial health deputy minister is part of Infoway. A committee, with three deputy health ministers and private and educational representatives, guides its operations. Infoway collaborates with partners regionally and nationally [4, 15].
**Malaysia** Government-wide digital agency mechanism.	Malaysia’s health system blends public and private services. Mandatory health insurance covers retirees, low-income individuals, and there’s also private insurance. State hospitals offer free care for the uninsured. It’s a two-tier system: the state provides tax-funded services, while the private sector has diverse providers [16, 17].	The Malaysian Administrative Modernization and Management Planning Unit (MAMPU) under the Prime Minister’s Office spearheads public service modernization. It coordinates with the MOH via an ICT Steering Committee. MAMPU sets digital health policies, guidelines, and approves related IT strategies. [18–22]
**England** Dedicated digital health agency mechanism + Health ministry mechanism.	The NHS, the world’s largest health service, covers England, Scotland, Northern Ireland and Wales, with each country setting its public health policy. The NHS in England is a state-run, tax-funded system, aiming to offer free health services to all [23–25].	The national IT strategy is overseen by the Department of Health and Social Care (DHSC), which also manages the digital health system’s budget. NHS England and NHS Impact determine the NHS IT strategy and procure via NHS Digital. The DHSC then funds NHS Digital, an autonomous entity that handles national IT operations and executes programs in collaboration with NHS England. With NHS restructuring favoring decentralization, Integrated Care Systems (ICSs) will soon bridge the NHS and local units, distributing budgets for digital investments serving local communities [26–32].

For instance, Canada’s decentralized model allows each province to set its health policy, leading to varied regional health policies. ‘Infoway’, a national non-profit agency, collaborates with representatives from every province, coordinating national health IT strategies and facilitating stakeholder cooperation. It also partners across sectors at both regional and national levels [2].

For Malaysia, the ‘Malaysian Administrative Modernization and Management Planning Unit (MAMPU)’ contributes to the government-wide digital agency mechanism. The MAMPU reports to the Prime Minister, and acts as a central agency coordinating with the Ministry of Health through the Information and Communication Technology Committee. This creates a central mechanism to oversee other agencies, ensuring unified direction [2].

#### Summary of in-depth interviews with stakeholders involved in the development of digital health systems

Most informants expressed that Thailand’s digital health governance should not be solely led by the MOPH. Their main argument is that health and IT services involve stakeholders beyond just MOPH, including the Ministry of Education, the Ministry of Digital Economy and Society, the Ministry of Higher Education, Science, Research and Innovation, university hospitals and the private sector, all of which the MOPH cannot directly manage and oversee.

Furthermore, informants cited the MOPH’s limited expertise in health information and communication technology, and the lack of expert personnel. These issues might hinder the country’s digital health adaptability to meet the current medical and public health needs. Therefore, most of the informants supported the ‘dedicated digital health agency mechanism’ or ‘government-wide digital agency mechanism’. Some preferred a mix of both, while others supported a combination of ‘dedicated digital health agency mechanism’ and ‘health ministry mechanism’.

In terms of leadership in the digital health governance, many interviewees believed it should be the Prime Minister or a designated Deputy Prime Minister, given their decision-making authority and cross-ministry coordination, should lead the country’s digital health governance. The interviews revealed the top factors affecting the country’s digital health governance, which are as follows:

1. Leadership: decisive support and decision-making for projects.
2. Inter-department collaboration: to reduce isolated and duplicated efforts, saving state expenses.
3. Data standards: crucial for data integration, facilitating benefits across health services, management, research, and medical and public health knowledge development.

### Stage 2

A total of 47 out of 101 participants (46.53%) answered the questionnaires. Majority (87%) had an education level higher than a bachelor’s degree. The background of the respondents includes health administrators (20.9%) and IT and science administrators (20.9%), IT and science practitioners (16.3%), health practitioners (14%) and others from various sectors including health insurance, budgeting and education (27.9%).

The degree of agreement regarding the **research finding** was 90.53% either strongly agreed (5) or agreed (4). Furthermore, the degree of agreement regarding the **recommendations** was 96.90% either strongly agreed (5) or agreed (4) (Table 3).

**Table 3:** The distribution of the respondents’ degree of agreement regarding the study findings and recommendations

	Number of respondent	Totally agree (5)	Agree (4)	Neutral (3)	Disagree (2)	Totally disagree (1)
**1.Research finding: factors essential for country to the success in development of digital health system**						
1.1 Senior-level government leaders (Leaderships) who recognize the necessity and importance of the digital health system.	47 (100%)	32 (68.1%)	11 (23.4%)	3 (6.4%)	1 (2.13%)	0 (0%)
1.2. A shared vision, a development framework and a strategy for developing a digital health system that stakeholders, both in the medical and public health and in the digital technology/ICT sectors, agree upon.	47 (100%)	33 (70.2%)	10 (21.5%)	4 (8.5%)	0 (0%)	0 (0%)
1.3 An effective governance mechanism: collaborative Governance mechanism.	47 (100%)	33 (70.2%)	9 (19.2%)	3 (6.4%)	2 (4.26%)	0 (0%)
1.4 Do you agree that all three factors mentioned above need to concurrently occur?	39 (100%)	27 (69.2%)	8 (20.5%)	2 (5.1%)	2 (5.1%)	0 (0%)
**2.Recommendations**						
2.1 The MOPH alone cannot manage digital health governance on its own but can lead in establishing a digital health governance mechanism.	46 (100%)	35 (76.1%)	11 (21.9%)	0 (0%)	0 (0%)	0 (0%)
2.2 The Ministry of Digital Economy and Society and the Ministry of Higher Education, Science, and Innovation must play a significant role in the governance mechanism.	46 (100%)	31 (67.4%)	13 (28.3%)	2 (4.4%)	0 (0%)	0 (0%)
2.3 The digital health governance mechanism should be collaboratively managed by all sectors and must consist of strong and consistent leaders.	46 (100%)	39 (84.8%)	7 (15.2%)	0 (0%)	0 (0%)	0 (0%)
2.4 The health insurance agency, health service providers and ICT businesses from both the public sector, private sector, and civil society must participate in the digital health governance process	46 (100%)	36 (78.3%)	10 (21.7%)	0 (0%)	0 (0%)	0 (0%)
2.5 There should be a central agency with authority and responsibility to drive the development of the country’s digital health system (Dedicated Digital Health Agency Mechanism).	46 (100%)	31 (67.4%)	11 (23.9%)	2 (4.4%)	2 (4.4%)	0 (0%)
2.6 Emphasis should be placed on having health information standards, such as the medical terminology standard SNOMED CT and health data linkage standard HL7 FHIR	36 (100%)	23 (63.9%)	11 (30.6%)	2 (5.6%)	0 (0%)	0 (0%)

In addition, there were 27 additional suggestions provided by the respondents including emphasizing the need to prioritize digital health nationally, ensure inter-organizational collaboration, involve the public and private sectors, adopt phased planning, empower agencies for digital health development, enhance expertise at all levels and address risks such as data security and operator safety.

## DISCUSSION AND CONCLUSIONS

This research identified three digital health governance formats: the MOH mechanism, the government-wide digital agency mechanism and the dedicated digital health agency mechanism [4], as illustrated in Table 1. To provide clear examples of each format, the researchers studied the digital health governance models of six countries, as shown in Table 2. These countries include Malaysia, the Philippines, Australia, England, the USA and Canada, to determine which model they align with or whether they have a hybrid model.

Interviews with 11 stakeholders in Thailand’s digital health system revealed a preference for the dedicated digital health agency and government-wide digital agency mechanisms. One limitation of the MOH mechanism is its inability to oversee external agencies.

Our findings resonate with the ITU-Broadband Commission Working Group on Digital Health report [4], emphasizing three crucial factors that must concurrently exist for successful digital health system development in a country:

1. Experienced leadership that understands the value of a digital health system.
2. A collective vision between both the medical and ICT/digital technology sectors.
3. An effective governance mechanism for the digital health system: the collaborative governance mechanism.

From our research, we recommend that Thailand’s Digital Health Governance mechanism should:

1. The MOPH should lead but not manage the governance solely.
2. Other ministries, including the Ministry of Digital Economy and Society and the Ministry of Higher Education, Science, and Innovation, should have significant roles.
3. Collaborative management by all sectors with consistent leadership is crucial.
4. Participation from health insurance agencies, service providers and ICT businesses across all sectors is essential.
5. A dedicated digital health agency should oversee the development of the digital health system.
6. Adhering to health information standards, such as SNOMED CT and HL7 FHIR, is crucial.

The evaluation of the research outcomes and policy recommendations by a diverse cohort of senior executives and representatives specializing in digital health from both public and private sectors provided their levels of concurrence. Quantitative analysis of the survey data indicates that in excess of 90% of participants expressed agreement or strong agreement with the presented findings and policy recommendations.

In 2023, the Thai cabinet formed the National Digital Health Committee, chaired by the Minister of Public Health, as a special committee under the auspices of the National Digital Economy and Society Commission, which is chaired by the Prime Minister. This was done through the Digital Development for Economy and Society Act of 2017.

It is worth mentioning the subject of digital health governance in India. The initiative for digital health in India was driven by the aim to achieve Universal Health Coverage, leading to the establishment of the National Health Authority (NHA), which is responsible for implementing the digital health ecosystem according to The Ayushman Bharat Digital Mission. The NHA is a fully functional autonomous entity governed by a Governing Board, chaired by the Union Minister of Health and Family Welfare and composed of high-level executives from both the Government of India and state governments. The digital health governance mechanism in India aligns with the dedicated digital health agency mechanism [33, 34].

Another country that has made rapid progress in digital health transformation is Indonesia. The Digital Transformation Office (DTO) was established at the Ministry of Health, along with the Digital Transformation Strategy 2021–2024. The DTO oversees the digital health transformation in many aspects, including planning, managing, conducting research, stakeholder consultation and centralizing development. The DTO collaborates with Pusdatin (The Center for Data and Information Technology of the Ministry of Health) in realizing the digital health transformation. The digital health governance mechanism of Indonesia is considered the Ministry of Health Mechanism [35, 36].

An Asian Development Bank’s report in 2018 [37], Transforming Health Systems Through Good Digital Health Governance, suggests key steps for establishing digital health governance. These steps include establishing consensus on governance aspects such as architecture and standards, selecting governance tools such as strategies and legal frameworks, and assembling a national steering committee led by ministries and supported by the private sector. The strategy emphasizes adopting a governance framework suitable for both central and subnational execution, monitoring performance to ensure accountability and updating the framework as technological advancements occur.

Digital health governance challenges are a common challenge in Asian Pacific countries that are transforming their national health information system and health systems. Comprehensive continuity of health care and effective health systems can only be achieved with good digital health governance [37].

### Study limitations

While valuable, this study had some limitations related to selection bias from the informants’ selection method and inadequate civil society representation. However, the digital health governance concept is not easy to conceptualize. It needs subjects who are working and familiar with digital health policy, management and implementation to provide insight information, and suggestions about the digital health governance model. Furthermore, this research is exploratory. Another limitation is the potentially outdated literature reviews due to the rapid change in digital health development globally. Although there are limitations, the output and results from stage 1 have been reviewed and confirmed by senior executives from stakeholders in digital health-related ministries, healthcare providers, professional agencies and private sectors in stage 2. This research is pioneering in shaping Thailand’s digital health governance policy.

## ACKNOWLEDGEMENTS

This research is part of a series of collaborative research projects undertaken by the working group to support the implementation of the Public Health Reform Plan (Revised Edition) in the area of Information Technology and Communication Technology for Health (Big Rock 6) under the Committee for National Health Reform. We employed the ChatGPT4 in the writing manuscript process.

## STUDY FUNDING

The responsibility for implementation has been assigned to the Center for Information Technology and Communication Technology, Office of the Permanent Secretary, Ministry of Public Health, with research funding support from the Health Systems Research Institute (HSRI).

## Data Availability

The data underlying this article cannot be shared publicly due to the privacy of individuals participating in the study.
